# Sex Differences in Sleep Duration among Older Adults with Self-Reported Diagnosis of Arthritis: National Health and Nutrition Examination Survey, 2009-2012

**DOI:** 10.1155/2018/5863546

**Published:** 2018-08-01

**Authors:** R. Constance Wiener, Alcinda K. Trickett Shockey, Christopher Waters

**Affiliations:** ^1^Dental Practice and Rural Health, P.O. Box 9448, Health Sciences Addition Room 104a, West Virginia University, Morgantown, WV 26506, USA; ^2^Department of Dental Hygiene, Robert C Byrd Health Sciences Center North, Room 1192A, West Virginia University, Morgantown, West Virginia 26506, USA; ^3^Department of Dental Research, P.O. Box 9448, Health Sciences Addition Room 106a, West Virginia University, Morgantown, WV 26506, USA

## Abstract

**Objective:**

Sleep is restorative, essential, and beneficial to health. Prevalences of some diseases have been associated with sleep duration. There are few studies in the literature on the relationship of sleep duration and arthritis stratified by sex in older adults. The purpose of this research is to investigate sleep duration among older adults in the United States who have self-reported diagnosis of arthritis.

**Methods:**

A cross-sectional study design was used. The data source was the National Health and Nutrition Examination 2009-2010 and 2011-2012. Self-reported diagnosis of arthritis and sleep duration were the variables of interest.

**Results:**

There were 4,888 participants, aged 50 years and above, of whom 41.6% self-reported having a diagnosis of arthritis, and 60.6% were female. Of the people who had a self-reported diagnosis of arthritis, 15.2% reported sleeping 2-5 hours as compared with 10.9% of the people who did not have a self-reported diagnosis of arthritis (*P* = .0004). In bivariate analysis of self-reported diagnosis of arthritis and sleep stratified by sex, there were significantly more people with self-reported diagnosis of arthritis who slept 2-5 hours for both women (*P* = 0.0192) and men (*P* = 0.0231). The overall relationship remained significant in adjusted overall logistic regression comparing for self-reported diagnosis of arthritis for 2-5 hours of sleep (with 6-7 hours of sleep as the reference) (odds ratio: 1.35 [95% CI: 1.08, 1.70; *P* = 0.0103]); however, when the data were stratified by sex, the association failed to reach significance.

**Conclusion:**

In this analysis of noninstitutionalized older adults in the United States, the prevalence of a self-reported diagnosis of arthritis was associated with shorter sleep duration in the overall analyses, but the association failed to reach significance when stratified by sex.

## 1. Introduction

Arthritis is a term that has been associated with more than 100 forms of joint pain. Several common types are osteoarthritis (also called degenerative arthritis); rheumatoid arthritis (an autoimmune disease); psoriatic arthritis; and gout. The most common form of joint disease, chronic joint pain, and disability is osteoarthritis [1, 2]. Worldwide, there are an estimated 9.6% of older men and 18% of older women who have osteoarthritis [3]. Degeneration of the cartilage at the joints due to damage from injury, overweight, use, or overuse occurs in weight-bearing joints over time. Knee/hip osteoarthritis was the 11^th^ reason in global disability determined from the Global Burden of Disease Study, 2010 [2]. Pain management/joint replacement are the available treatment options for osteoarthritis [4].

Rheumatoid arthritis occurs when the immune system attacks a person's joints. Joint pain and damage can occur with the associated inflammation. The inflammation is thought to be related to tumor necrosis factor and interleukin-1 causing the immune system to overreact [5]. In addition to painful joints, people with rheumatoid arthritis often feel tired.

In the US psoriatic arthritis occurs in about 11% of people with psoriasis [6]. The prevalence of psoriasis is 0.25% in the US [6]. The skin and joints become inflamed with psoriatic arthritis.

Gout occurs when uric acid crystals concentrate in a joint, typically the big toe. The pain is often associated with excessive drinking, drugs, or stress. Untreated, gout can progress to affect other joints and the kidneys.

Management of the many types of joint pain is specific to the unique causes. Unfortunately, there is a great deal of confusion about arthritis in general when people are not specific about the type of arthritis being discussed.

However, arthritic symptoms from any of the many types of arthritis impact quality of life, trend to disability, and are associated with high economic burdens [7]. Arthritic symptoms are influenced and modified by, as well as influence and modify, many factors including sleep. It is theorized that sleep is restorative, protective, instinctive, conservative, and necessary for a good quality of life, both physically and psychologically [8]. As individuals grow older, sleep duration decreases while waking frequency increases [9, 10]. An individual's sex and health status are two major factors that may account for the variation in sleep patterns observed in studies involving sleep and age [10]. In general, sleep duration for healthy adults is approximately 7-9 hours [9, 11].

### 1.1. Sleep Studies

For* older *adults, researchers of a study of 1,026 older adult participants in Paris [12], as well as a study of 66,478 adult participants in Korea [9], indicated a median night-time sleep duration of 7 hours. Researchers using the Behavioral Risk Factor Surveillance System, 2014, reported that more than a third of US adults were sleeping fewer than 7 hours each night [13, 14]. Short sleeping* older* adults were identified by researchers as sleeping less than 6 hours per night and long sleepers as sleeping more than 9 hours per night in a study on global aging and health in adults who were aged 50 years and above [15]. In the United States, 37.3% of adults aged 45-64 years and 26.3% of adults older than 65 years reported a sleep duration of less than 7 hours [13]. There were 62.7% and 73.7%, respectively, who reported more than 7 hours of sleep [13].

### 1.2. Arthritis and Sleep Studies

Individuals with arthritis are thought to have a bidirectional relationship of arthritic symptoms and sleep duration [16]. Fatigue in participants with rheumatoid arthritis (RA) was shown to be associated with poor sleep quality in a small sample (*n* = 158) of older adults in the United States [17], and in a sample (*n* = 986) of participants from Norway [18]. Previous researchers have indicated that certain disease prevalences were affected by sleep duration. Sleep and arthritis have been investigated, but few researchers have evaluated subgroups. The researchers conducting analyses involving subgroups by* race/ethnicity* in the United States found no differences in total sleep time in older adults with osteoarthritis (*n* = 124) [19]. Similar research of older adults with arthritis based upon sex has been limited to a study using Korea National Health and Nutrition Examination Survey 2010-2013 data, in which researchers did find a relationship of sleep duration and osteoarthritis [16].

The purpose of this research is to investigate sleep duration by sex among older adults in the United States who have self-reported diagnosis of arthritis using data from the National Health and Nutrition Examination Survey, 2009-2012. The null hypothesis is that the odds for sleep duration for people with self-reported diagnosis of arthritis are equal to the odds for sleep duration for people without self-reported diagnosis of arthritis, stratified by sex with an a priori *P* value established at < 0.05.

The Krieger Ecosocial epidemiological theory was used for the theoretical framework of the study. In the theory many factors should be included when attempting to determine associations with the embodiment of an outcome/condition/disease including those which are biological, behavioral, and physical/environmental, and those related to accountability and agency within societal and ecosystem levels from the individual, to households, groups, regions, and nations and globally over the course of life [20]. In this framework, sleep duration is expected to have a biological difference with sex hormones and the reproductive system contributing as factors, as well as sex, differences in work, recreation, lifestyle, habits, stress, psychosocial factors, healthcare access, etc. [8].

## 2. Materials and Methods

The National Health and Nutrition Examination Surveys (NHANES) from years 2009-2010 and 2011-2012 (from which the data for this study were extracted) were conducted under two National Center for Health Statistics Research Ethics Review Board protocols: (1) Protocol Continuation of 2005-06 (for NHANES 2009-2010) and (2) Protocol 2011–17 (for NHANES 2011-2012). The West Virginia University Institutional Review Board provided ethics acknowledgement of this secondary data analysis study of the existing, publicly available, deidentified NHANES data as being nonhuman subject research under Protocol Number 1803045894.

### 2.1. Study Design, Data Source, and Data Availability

The researchers used the combined NHANES, 2009-2010 and 2011-2012, data source, as mentioned above. This study had a cross-sectional, observational, epidemiological research design, based upon the study design of Jung et al. [16]. Guidelines from the Strengthening the Reporting of Observational Studies in Epidemiology (STROBE) were followed for this study.

The researchers who completed the NHANES used a stratified, multistage probability sampling design for the NHANES 2009-2012 surveys. The participants were noninstitutionalized United States civilians with an oversampling of minority subgroups. The NHANES data for this study are publicly available. Additional information about the NHANES and access to the data are available at https://wwwn.cdc.gov/nchs/nhanes/Default.aspx.

### 2.2. Study Sample

The inclusion criteria for participants in this study of self-reported diagnosis of arthritis and sleep duration were that participants were aged 50 years or above, and that they had no missing data on self-reported diagnosis of arthritis status, number of hours that are routinely slept, sex, race/ethnicity, education, smoking, insurance, body mass index, and self-perceived health status.

### 2.3. Measures: Main Variables of Interest

The study's main variables were self-reported diagnosis of arthritis and sleep duration. The self-reported diagnosis of arthritis variable was created from a question posed to NHANES participants about their arthritis status (yes/no). The question was: “Has a doctor or other health professional ever told you that you had arthritis?” [21]. Sleep duration/the number of hours of sleep was created from a question posed to NHANES participants about the number of hours routinely slept by the participants: “How much sleep do you get (hours)?” [21].

### 2.4. Measures: Covariables

Other variables for the study were sex (male; female), race/ethnicity (non-Hispanic White, non-Hispanic Black, Mexican-American, and other), age (50 to less than 60, 60 and above), smoking status (current smokers, former smokers, and never smokers), body mass index (less than 25, 25 to less than 30, and 30 and above), insurance (insured, not insured), and self-perception of health (excellent/very good, good, and fair/poor).

### 2.5. Statistical Analyses

The statistical tests used in this study were Chi square tests for bivariate associations between self-reported diagnosis of arthritis and the other variables. Unadjusted logistic regression analyses were used to determine the association of arthritis and the sleep variable (1) in overall analyses; (2) in subgroup analysis by females; and (3) in the subgroup analysis by males. The adjusted logistic regression analyses included the other variables listed above. Sampling weights, primary sampling units, domain, and strata were accounted for in the data analyses. The analyses were completed with the Statistical Analysis System Software (SAS® version 9.3, SAS Institute, Inc., Cary, NC, USA).

## 3. Results

### 3.1. Study Sample Description

This study included 4,888 participants. There were 76.7 (weighted) % non-Hispanic White participants, 9.9% non-Hispanic Black participants, 4.2% Mexican-American participants, and 9.2% other participants. More participants were aged 60 years and older, had more than a high school education, and slept 6-7 hours (57.7%, 57.8%, and 51.4%, respectively). There were 41.6% (*n* = 2,132) of the participants who self-reported a diagnosis of having arthritis. Of the participants with a self-reported diagnosis of arthritis, 60.6% were female. Sample details are presented in Table 1.

### 3.2. Bivariate Associations with Self-Reported Diagnosis of Arthritis

Also presented in Table 1 are the bivariate associations of self-reported diagnosis of arthritis and the other variables. For the main variable of interest, hours of sleep, 15.2% of participants with a self-reported diagnosis of arthritis reported sleeping 2-5 hours as compared with 10.9% of the people who did not self-report a diagnosis of arthritis (*P* = .0004). In bivariate analysis of self-reported diagnosis of arthritis and sleep stratified by sex, there were significantly more people with self-reported diagnosis of arthritis who slept 2-5 hours for both women (*P* = 0.0192) and men (*P* = 0.0231).

Other significant overall bivariate associations with self-reported diagnosis of arthritis were with race/ethnicity, age, education, smoking, body mass index, insurance, and perceived health status. More people who were non-Hispanic Black (44.8%) and non-Hispanic White (42.2%) self-reported a diagnosis of arthritis than people who were Mexican-American (35.6%) (*P* = 0.0013). The race/ethnicity relationship with self-reported diagnosis of arthritis was not significant when stratified by sex for males. It remained significant; for females there were 25.6% of Mexican-American females who self-reported a diagnosis of arthritis compared with 36.0% of non-Hispanic Black women and 36.1% of non-Hispanic White women (*P* = 0.0115).

More adults aged 60 years and above, as compared with adults aged 50-59, self-reported diagnosis of arthritis (48.4% as compared to 32.3%, *P* < .0001). The results remained significant concerning age and self-reported diagnosis of arthritis in subgroup analysis for females, as well as for males (*P* < .0001).

People with a high school education or less were more likely to report a diagnosis of arthritis (44.5%) as compared with people having more than a high school education (39.4%, *P* = 0.0044). The relationship remained significant concerning education and self-reported diagnosis of arthritis in subgroup analysis for females (*P* = 0.0143) but failed to reach statistical significance for males (*P* = 0.7210).

There were 38.1% of never smokers who reported a diagnosis of arthritis, as compared with 46.2% of former smokers and 42.2% of current smokers (*P* = 0.0012). The relationship remained significant concerning smoking status and diagnosis of arthritis in subgroup analysis for females (*P* = 0.0055) and males (*P* < .0001).

People with a body mass index of 30 and above and people with a body mass index of 25-29 were more likely to report a diagnosis of arthritis (38.6% and 36.1%, respectively) as compared with people with a body mass index of 24 or less (25/4%, *P* < .0001). The relationship remained significant concerning body mass index and self-reported diagnosis of arthritis for both females (*P* < .0001) and males (*P* = .0002).

People who did have insurance were more likely to self-report a diagnosis of arthritis as compared with people who did not have insurance (43.0% versus 29.7%, *P* = 0.0002). The relationship failed to remain significant for females. There were 37.0% of males with insurance and 19.2% of males without insurance who self-reported a diagnosis of arthritis (*P* < .0001).

There were 55.8% of people with a self-perception of fair/poor health who reported a diagnosis of arthritis as compared with 45.5% of people with a self-perception of good health and 31.4% of people with a self-perception of excellent/very good health who reported arthritis (*P* < .0001). The relationship of perceived health status and self-reported diagnosis of arthritis remained significant in subgroup analysis for males and females (*P* < .0001).

### 3.3. Results of Logistic Regression Analyses

Overall unadjusted and adjusted logistic regression analyses and sex subgroup unadjusted and adjusted logistic regression analyses are presented in Table 2. The overall unadjusted odds ratio (OR) of having self-reported a diagnosis of arthritis and sleeping 2-5 hours was 1.49 [95% Confidence interval (CI): 1.18, 1.87; *P* < .0001] as compared with sleeping 6-7 hours. It remained significant in the adjusted model in which the adjusted OR was 1.35 [95% CI: 1.08, 1.70; *P* = 0.0103].

Stratified by the female sex, the unadjusted OR of having self-reported diagnosis of arthritis and sleeping 2-5 hours was 1.56 [95% CI: 1.14, 2.13; *P* = 0.0190] as compared with sleeping 6-7 hours. However, the relationship was attenuated and failed to reach significance in the adjusted model in which the adjusted OR was 1.29 [95% CI: 0.94, 1.77; *P* = 0.1132].

Stratified by the male sex, the unadjusted OR of having self-reported diagnosis of arthritis and sleeping 2-5 hours was 1.41 [95% CI: 0.98, 2.02; *P* = 0.0622] as compared with sleeping 6-7 hours. In the adjusted model, the adjusted OR was 1.40 [95% CI: 0.94, 2.08; *P* = 0.0977]. Both unadjusted and adjusted failed to reach significance.

## 4. Discussion

Previous researchers have reported that there are certain disease prevalences and outcomes that were found to be associated with hours of sleep. For example, Katsagoni et al. reported that having a healthy diet-optimal sleep lifestyle was inversely associated with liver stiffness and insulin resistance [22]. Yeo et al. reported that mortality from cardiovascular disease and respiratory disease increased in participants who slept ≤5 hours as compared with participants who slept 7 hours [23]. Choi et al. reported an increase in atopic dermatitis and asthma in females with sleep duration ≤5 hours compared with participants who slept 7 hours [24]. And Jung et al., who evaluated data from patients who had knee or hip joint radiography, reported that male patients who slept 0-3 hours and 4-5 hours had adjusted odds ratio of 2.28 and 1.38 for osteoarthritis, respectively, as compared with patients who slept 6-7 hours; and, for the women patients, the respective adjusted odds ratios for osteoarthritis were 1.63 and 1.26 [16].

The researchers for this study examined the relationship between self-reported diagnosis of arthritis and sleep duration in older adults, aged 50 years and above. The most common sleep duration category was 6-7 hours. Few participants (12.7%) reported limited sleep (2-5 hours per night). The self-reported diagnosis of arthritis was high (41.6%) in this group of older adults. Overall, there was a relationship of limited sleep and self-reported diagnosis of arthritis (adjusted odds ratio: 1.35 [95% CI: 1.08, 1.70; *P* = 0.0103]). Adjusted odds ratios stratified by male or female sex failed to reach significance.

### 4.1. Similar Studies in Support of or in Contrast to This Study's Results

Jung et al. conducted a study in Korea and reported the adjusted odds ratios for osteoarthritis based upon hours of sleep for men (3.22) and women (2.05), both of which were statistically significant [16]. Although the adjusted overall relationship of self-reported diagnosis of arthritis (the variable used in this study) was significant and positive in this study (1.35), the adjusted odds ratios for females (1.29) and males (1.40), although positive, were not statistically significant. Jung et al. study's variable was osteoarthritis based upon a radiographic criterion of a knee/hip joint Kellgren-Lawrence grade of 2 or above, whereas self-reported diagnosis of arthritis was the variable used in this study.

The findings of this study were similar to a study conducted by Dai and Hao in 2017 who used 2014 Behavioral Risk Factor Surveillance System data and found significant associations of very short sleep duration, less than 5 hours, and arthritis, but failed to find significance in sleep duration and sex [14]. Researchers of a recent study of 2,682 people, aged 45 years and above, with/without symptomatic osteoarthritis of the knee or hip in Johnston County, NC, US, reported that 55% of all individuals in the study had insufficient sleep and the individuals with symptomatic osteoarthritis had an unadjusted odds ratio of 1.35 (95% CI 1.12–1.62) for insufficient sleep as compared with individuals who did not have symptomatic osteoarthritis of the knee or hip [25]. The overall unadjusted odds ratio for this current study is in agreement with the Johnston County, NC, US, study's overall results which was one of the first to investigate sleep disturbances in people with/without osteoarthritis [25].

Researchers in Egypt used the Pittsburgh Sleep Quality Index (PSQI) and compared men and women with/without arthritis and reported that “poor sleepers” (who were identified using PSQI definitions) were more likely to have arthritis, but no significant association between the PSQI and gender was reported [26]. These overall results of no differences in sleep duration between men and women are similar to the results of a meta-analysis of sleep in adults aged 58 years and above [8].

### 4.2. Study Strengths and Limitations

A study strength was the use of a large, valid, consistently running, and nationally representative study of United States residents. As this was a large study, many factors known to influence arthritis and sleep could be included in the analyses to allow for adjusted odds ratios and the influence of other factors. Another strength is the use of categories consistent with previous research [16] so that any future meta-analysis would have greater validity. The use of categories, rather than specific number of hours, also helps in reducing the potential for self-reporting errors.

A characteristic of cross-sectional epidemiological studies (rather than a specific limitation) is that causation cannot be inferred. This does limit the results of this or any cross-sectional study in terms of assessing causation. Therefore, epidemiological studies are often followed by clinical studies to document temporal associations.

Another limitation of this study was the use of self-reports for both diagnosis of arthritis and sleep duration. Self-reports can be influenced by poor recall of the participants, or by a desire to respond to the interviewer in a socially acceptable manner (social desirability bias), or by misunderstanding a question. The question posed to the participants about arthritis was a general question as to if he or she had received a diagnosis for arthritis. The type of arthritis was not specified. There is a potential that the participant understood the question to only mean osteoarthritis or rheumatoid arthritis. However, self-reports are often used in epidemiological studies, and the NHANES researchers have developed their surveys to be valid and reliable over the years since the NHANES began in 1971.

## 5. Conclusion

Our results, and those of other researchers, highlight the importance of adequate sleep duration. In this analysis of noninstitutionalized older adults in the United States, the prevalence of a self-reported diagnosis of arthritis was associated with shorter sleep duration in the overall analyses, but the association failed to reach significance when stratified by sex.

## Figures and Tables

**Table 1:** Sample characteristics according to self-reported diagnosis of arthritis, *n* = 4,888, ages 50 years and above, from National Health and Nutrition Examination Survey, 2009-2012.

**Category**	**Total**		**Total**				**P** **-value**	**Female (** **n** = 2455**)**				**P** **-value**	**Male (** **n** = 2433**)**				**P** ** value**
			**Arthritis**		**no Arthritis**			**Arthritis**		**no Arthritis**			**Arthritis**		**no Arthritis**		
	**n**	**wt**%	**n**	**wt**%	**n**	**wt**%		**n**	**wt**%	**n**	**wt**%		**n**	**wt**%	**n**	**wt**%	
**Sex**																	
Female	2455	52.9	1260	47.6	1195	52.4	**<.0001**										
Male	2433	47.1	872	34.8	1561	65.2											
**Race/Ethnicity**																	
NHW	2281	76.7	1092	42.2	1189	57.8	**0.0013**	621	47.7	518	52.3	.0848	471	36.1	671	63.9	**.0115**
NHB	1134	9.9	514	44.8	620	55.2		299	51.9	270	48.1		215	36.0	350	64.0	
Mex-Am	584	4.2	214	35.6	370	64.4		139	46.8	145	53.2		75	25.6	225	74.4	
Other	889	9.2	312	35.3	577	64.7		201	42.4	262	57.6		111	27.1	315	72.9	
**Age (years)**																	
50-59	1590	42.3	518	32.3	1072	67.7	**<.0001**	310	37.3	475	62.7	**<.0001**	208	27.2	597	72.8	**<.0001**
60 and above	3298	57.7	1614	48.4	1684	51.6		950	54.4	720	45.6		664	41.0	964	59.0	
**Education**																	
≤HS graduate	2593	42.2	1185	44.5	1408	55.5	**.0044**	721	52.4	586	47.6	**.0143**	464	35.4	822	64.6	.7210
>HS	2295	57.8	947	39.4	1348	60.6		539	44.0	609	56.0		408	34.3	739	65.7	
**Smoking**																	
Current	792	15.3	337	42.2	455	57.8	**.0012**	173	56.6	126	43.4	**.0055**	164	31.9	329	68.1	**<.0001**
Former	1681	35.0	810	46.2	871	53.8		371	51.2	277	48.8		439	42.1	594	57.9	
Never	2415	49.7	985	38.1	1430	61.9		716	43.9	792	56.1		269	28.6	638	71.4	
**Body Mass Index**																	
0-24	1213	25.4	441	37.3	772	62.7	**<.0001**	270	41.3	345	58.7	**<.0001**	171	31.6	427	68.4	**.0002**
25-29	1733	36.1	680	36.6	1053	63.3		354	42.6	407	57.4		326	31.2	646	68.8	
30 and above	1942	38.6	1011	49.0	931	51.0		636	56.1	443	43.9		375	40.5	488	59.5	
**Hours of sleep**																	
2-5	800	12.7	402	49.8	398	50.2	**.0004**	238	57.0	170	43.0	**.0192**	164	41.9	228	58.1	**.0231**
6-7	2339	51.4	980	40.0	1359	60.0		579	45.9	598	54.1		401	33.9	761	66.1	
8-9	1598	33.6	671	39.9	927	60.0		399	45.9	398	54.1		272	32.3	529	67.7	
10 and above	151	2.4	79	53.9	72	46.1		44	56.8	29	43.2		35	50.0	43	50.0	
**Insurance**																	
Yes	4171	89.1	1924	43.0	2247	57.0	**.0002**	1131	48.1	986	51.9	.2814	793	37.0	1261	63.0	**<.0001**
No	717	10.9	208	29.7	509	70.3		129	42.1	209	57.9		79	19.2	300	80.8	
**Perceived Health Status**																	
Excellent/v good	1572	42.6	543	31.4	1029	68.6	**<.0001**	307	35.7	470	64.3	**<.0001**	236	26.2	559	73.8	**<.0001**
Good	1884	37.4	820	45.5	1064	54.5		488	52.5	459	47.5		332	38.2	605	61.8	
Fair/Poor	1432	29.0	769	55.8	663	44.2		465	65.0	266	35.0		304	45.6	397	54.4	

Hours of sleep were 2 to less than 5.5, 5.5 to less than 7.5, 7.5 to less than 9.5, and 9.5 and above. Age was 50 to less than 60 and 60/above. Body mass index was 0 to less than 25, 25 to less than 30, and 30/above. wt, weighted; NHW, non-Hispanic White; NHB, non-Hispanic Black; Mex-Am, Mexican-American; ≤, less than or equal to; HS, high school; >, greater than; and v, very.

**Table 2:** Odds ratios (ORs), adjusted odds ratios (AORs), and 95% confidence intervals (CIs) of sleep from logistic regression on self-reported diagnosis of arthritis National Health and Nutrition Examination Survey, 2009-2012, data.

	**Unadjusted Logistic Regression**		**Adjusted Logistic Regression**	
	**OR [95**%** CI]**	**P** ** value**	**AOR [95**%** CI]**	**P** ** value**
**OVERALL **(*n* = 4,888)				
Sleep				
2 -5 hours	**1.49 [1.18, 1.87]**	**<.0001**	**1.35 [1.08, 1.70]**	**0.0103**
6-7 hours	reference		reference	
8-9 hours	0.99 [0.82, 1.20]	0.9475	0.90 [0.74, 1.10]	0.3008
10 hours and above	**1.75 [1.17, 2.62]**	**0.0077**	1.25 [0.77, 2.04]	0.3515
**FEMALE ONLY **(*n* = 2,455)				
Sleep				
2 -5 hours	**1.56 [1.14, 2.13]**	**0.0190**	1.29 [0.94, 1.77]	0.1132
6-7 hours	reference		reference	
8-9 hours	1.00 [0.75, 1.32]	0.9796	0.96 [0.70, 1.31]	0.7730
10 hours and above	1.55 [0.88, 2.73]	0.1273	1.14 [0.57, 2.29]	0.7085
**MALE ONLY **(*n* = 2,433)				
Sleep				
2 -5 hours	1.41 [0.98, 2.02]	0.0622	1.40 [0.94, 2.08]	0.0977
6-7 hours	reference		reference	
8-9 hours	0.93 [0.72, 1.21]	0.5807	0.84 [0.64, 1.09]	0.1759
10 hours and above	**1.95 [1.03, 3.67]**	**0.0407**	1.47 [0.71, 3.01]	0.2870

Hours of sleep were 2 to less than 5.5, 5.5 to less than 7.5, 7.5 to less than 9.5, and 9.5 and above. Adjusted for age (50 years to less than 60, 60 and above), race/ethnicity (non-Hispanic White, non-Hispanic Black, Mexican-American, and other), education (high school graduate or less, more than High School), smoking (current, former, and never), insurance (insured, not insured), self-perception of health (excellent/very good, good, and fair/poor), and body mass index (0 to less than 25, 25 to less than 30, and 30 and above).

## Data Availability

Previously reported and published data from the National Health and Nutrition Examination Survey, 2009-2012, were used in this study and are available at https://wwwn.cdc.gov/nchs/nhanes/continuousnhanes/default.aspx?BeginYear=2011. The dataset is cited at relevant places within the text as reference [21].
